# Supplementary material for: Epigenetic Modifications of the Liver Tumor Cell Line HepG2 Increase Their Drug Metabolic Capacity
Source: Int J Mol Sci. 2019 Jan 16;20(2):347. doi: 10.3390/ijms20020347 (PMC6358789; doi:10.3390/ijms20020347)
Supplement: Supplementary file 1 [file ijms-20-00347-s001.pdf]

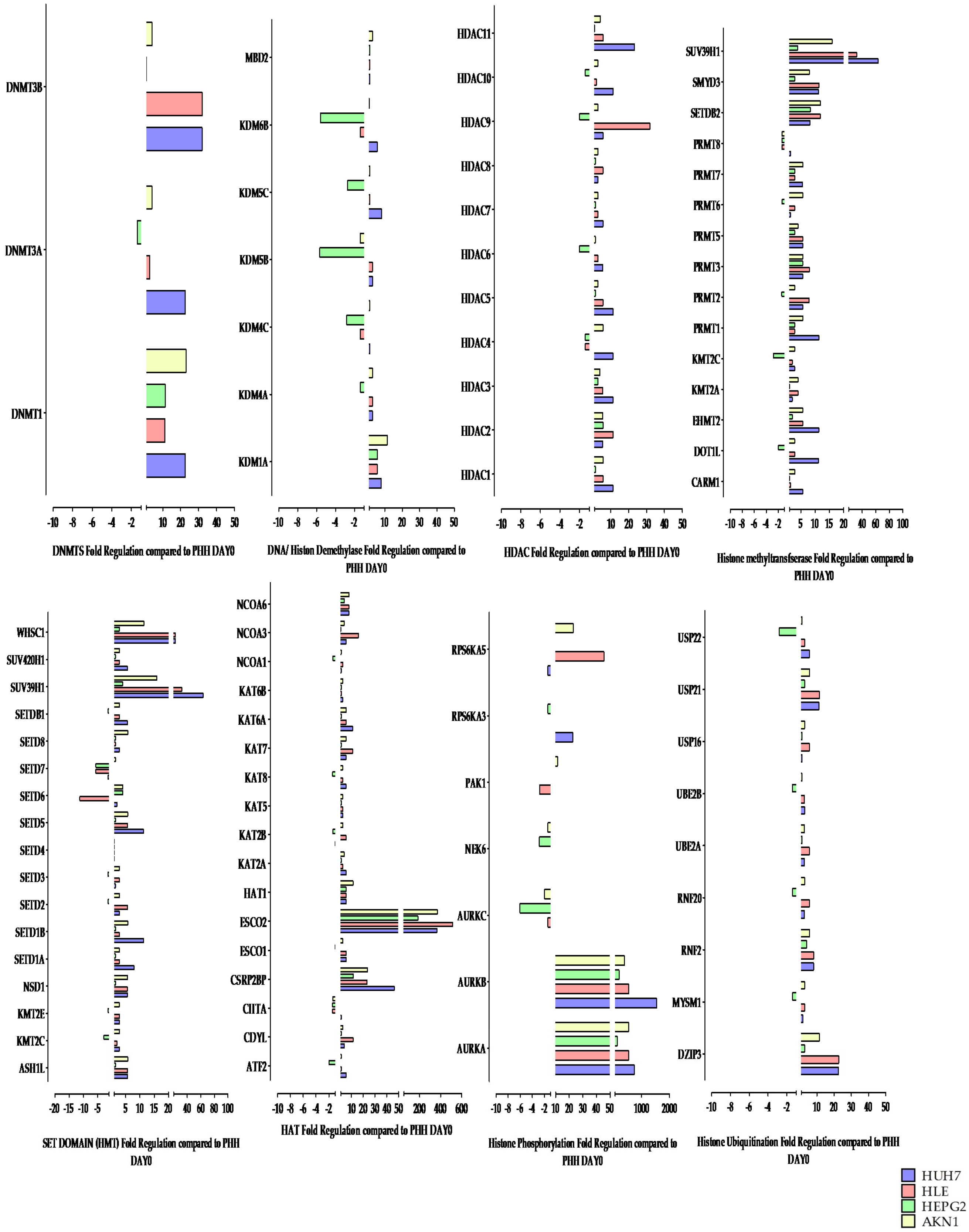

Figure 1. Summary of all measured genes in the cell lines AKN1, HLE, Huh7 and HepG2 compared to PHH by using Chromatin Modification PCR Array

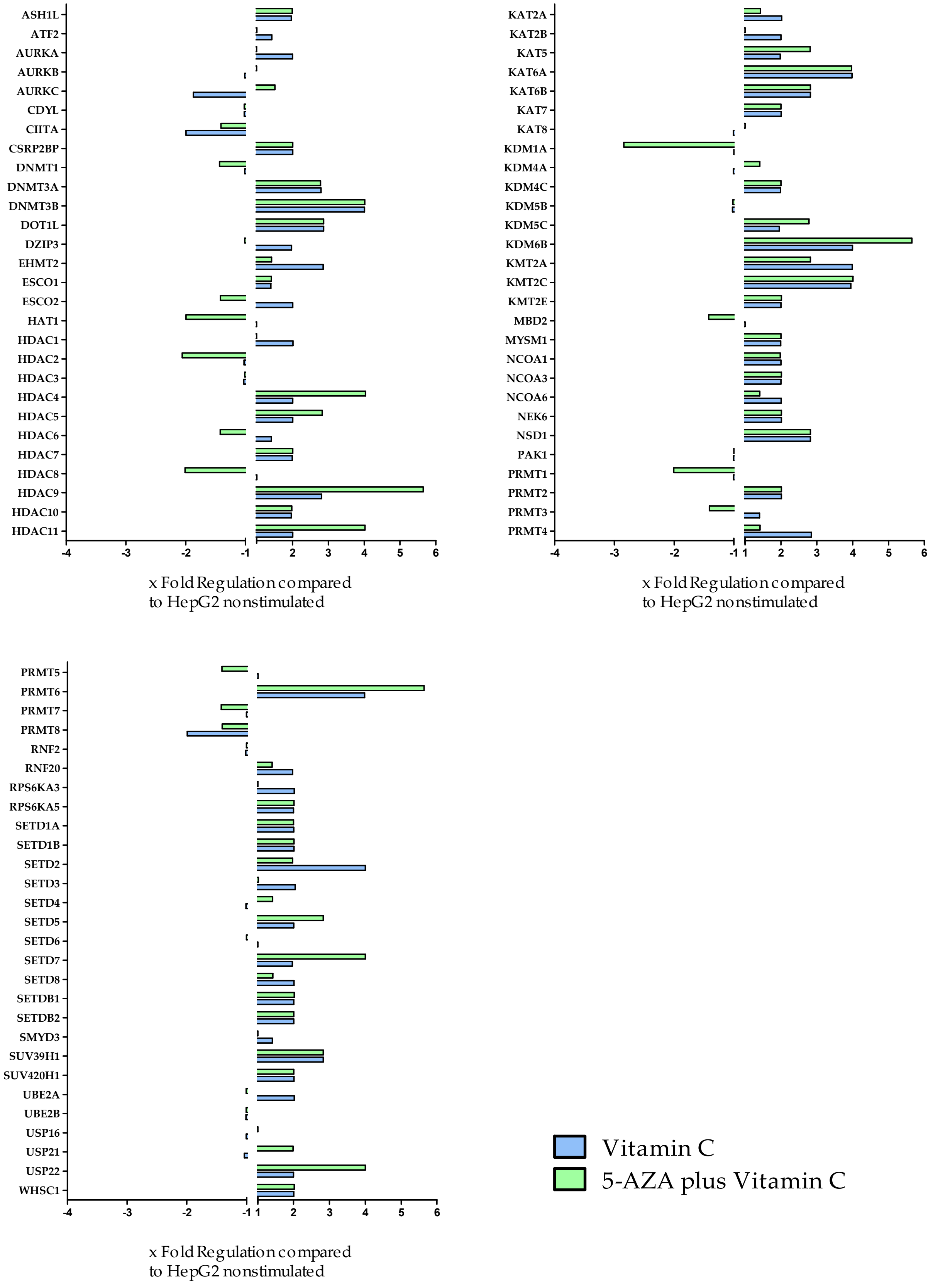

Figure 2. Summary of all measured genes in HepG2 stimulated with Vitamin C alone or with 5-AZA and Vitamin C in combination, compared to untreated control by using Chromatin modification PCR Array
